# Association of cardiovascular health with COPD (NHANES 2007-2020): mediating potential of lean body mass

**DOI:** 10.3389/fendo.2025.1539550

**Published:** 2025-04-04

**Authors:** Ruoyu Gou, Xiaoyu Chang, Danni Dou, Xin Meng, Ling Hou, Lingqin Zhu, Wei Tuo, Guanghua Li

**Affiliations:** ^1^ School of Public Health, Ningxia Medical University, Yinchuan, China; ^2^ School of Basic Medical Sciences, Ningxia Medical University, Yinchuan, China; ^3^ People’s Hospital of Ningxia Hui Autonomous Region, Yinchuan, Ningxia, China

**Keywords:** COPD, life’s crucial 9 (LC9), lean body mass (LBM), NHANES, mediating potential

## Abstract

**Background:**

Chronic Obstructive Pulmonary Disease (COPD) is a major global health concern, with lifestyle factors playing a crucial role in its prevention. This study aims to explore the relationship between Life’s Crucial 9 (lc9) scores and COPD odds, and to assess the mediating potential of lean body mass (LBM) in this association.

**Methods:**

This study used cross-sectional study to assess the association between lc9 score and COPD using data from the National Health and Nutrition Examination Survey (NHANES) from 2007 to 2020. Weighted multivariate regression analyses were performed to examine lc9 score on the odds of COPD after adjusting for confounders. The models were adjusted for age, gender, race/ethnicity, Marital status, education level, Family income-to-poverty ratio, LBM and Alcohol consumption status. The discrimination ability of lc9 on COPD odds was evaluated using (ROC) curve. Mediation analysis was used to investigate the mediating potential of LBM between lc9 and COPD odds. Subgroup analyses and interaction assessments were also performed.

**Results:**

In Model 2, the results showed that for every 10-point change in the lc9 score, the odds of developing COPD decreased. The OR (95% CI) in the Moderate and High groups were OR = 0.37; 95% CI: 0.23, 0.59 and OR = 0.16; 95% CI: 0.09, 0.27 (*P for trend* < 0.001), respectively. In addition, the results for quartile subgroups were Q3, OR = 0.58; 95% CI: 0.42, 0.81), Q4, OR = 0.24; 95% CI: 0.16, 0.36) and *P for trend* < 0.001. This relationship was consistent across the total population, subgroup analyses, and sensitivity analyses. There was a nonlinear relationship between lc9 score and odds of COPD (*P for Nonlinear* = 0.022). The lc9 reduced the odds of COPD by increasing LBM. The lc9 is an suggestive predictor of COPD odds association.

**Conclusions:**

Higher LC9 scores, particularly when accompanied by increased LBM levels, showed significant associations with reduced COPD risk in cross-sectional analyses.

## 1 Introduction

Chronic obstructive pulmonary disease (COPD) is a heterogeneous lung disease characterized by persistent respiratory symptoms and airflow obstruction caused by abnormalities in the airways or alveoli (1). With high morbidity and disability rates, COPD has become the third leading cause of death globally (2), In 2019, there were 212 million cases of COPD worldwide, posing a serious burden on patients’ quality of life and public health (3, 4). Studies have shown that the etiology of COPD is complex, and in addition to genetic factors, environmental factors, especially lifestyle factors, play a crucial role in its development and progression (5, 6). Traditionally, smoking has been recognized as a major odds factor for COPD (7), but increasing evidence suggests that other lifestyle factors, such as an unhealthy diet (8, 9), physical inactivity (10), and obesity (11), may also be closely associated with the development of COPD. While COPD is treatable, it is not completely curable.

The American Heart Association introduced “Life’s Simple 7” and expanded it to “Life’s Essential 8” (LE8) and “Life’s Crucial 9” (lc9) to promote Cardiovascular health (12–14). The “lc9” builds on the American Heart Association’s “le8” by adding psychological health as an extra component (12). The nine elements of lc9 are Eat Better, Be More Active, Manage Weight, Manage Blood Sugar, Control Cholesterol, Manage Blood Pressure, Quit Tobacco, Get Healthy Sleep, and Address Psychological Health (15). These lifestyle indicators’ protective benefits for cardiovascular health are widely acknowledged in academia, and they have been demonstrated to lessen the odds of a variety of chronic illnesses. While the associations between LC9 scores and cardiometabolic conditions are well documented (16), their linkages to respiratory system disorders—especially COPD remain systematically underexplored in population-based studies.

LBM (LBM) is a reliable body measurement tool that incorporates data such as height, age, weight, and waist circumference (17). In recent years, it has gradually attracted the attention of researchers. LBM is not only associated with metabolic health, but it also plays a significant role in the prevention and management of chronic diseases (18). According to studies, those with more LBM typically have superior lung function, and they may also be at a lower odds of developing respiratory disorders (19, 20), particularly COPD. LBM has thus been identified as a possible protective factor against developing and progression of COPD. However, the potential mediating of LBM between lc9 and COPD has not been thoroughly investigated in the literature to date.

The aim of this study was to explore the relationship between lc9 and COPD using the National Health and Nutrition Examination Survey (NHANES) database, as well as to analyze the potential mediating of LBM in it. We hypothesize that high levels of lc9 score are associated with a lower odds of COPD, while LBM may play an suggestive mediating role in this relationship. This study analyzes the link between lc9, LBM, and COPD, providing a scientific basis for prevention and management. It also contributes to a better understanding of the role of LBM and lifestyle factors in chronic disease prevention and control, and serves as a reference for public health intervention strategies.

## 2 Methods

### 2.1 Study design and population

**Figure 1:**
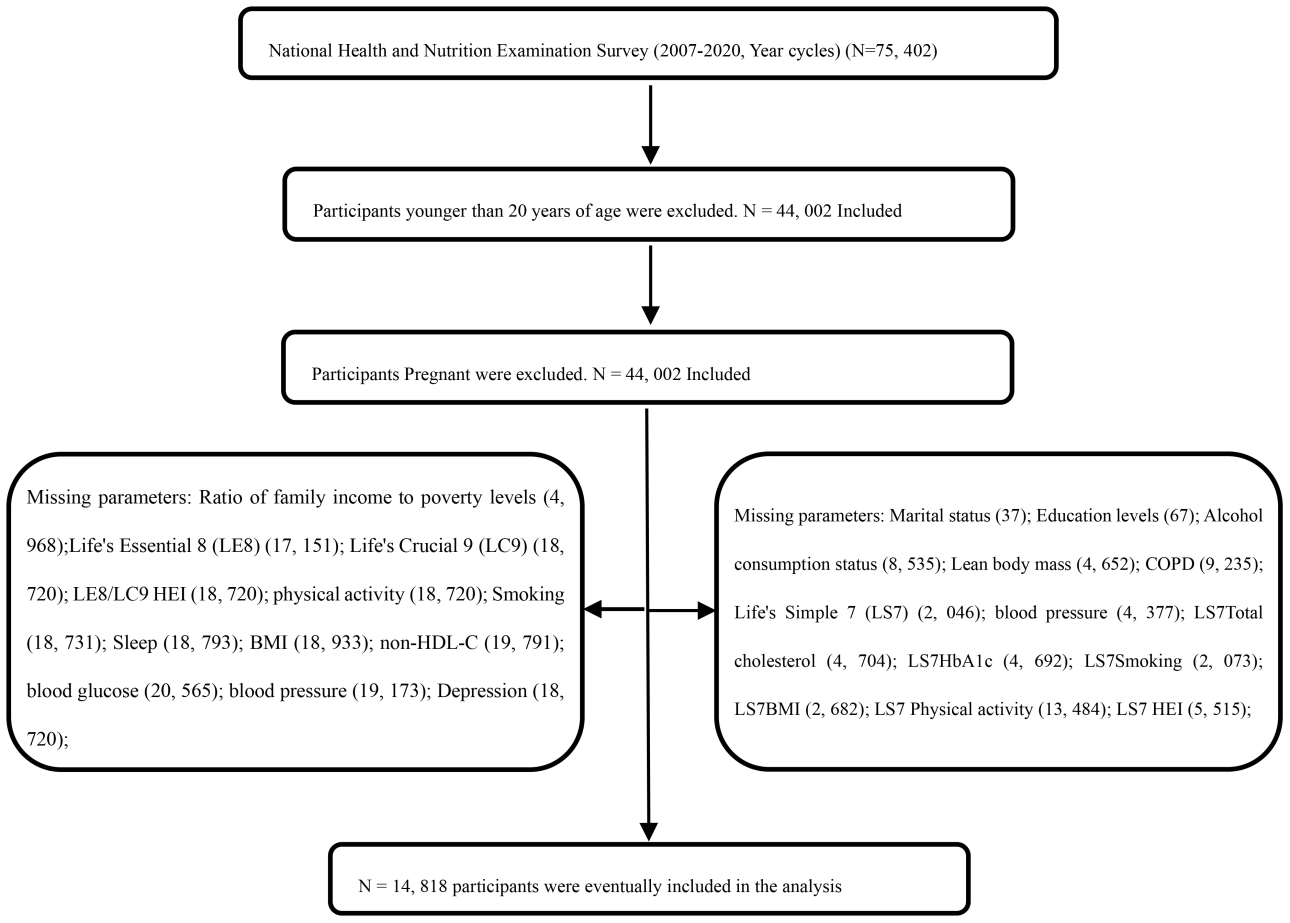
Flow chart of the screening process for the selection of the study population.

In this study, data from the NHANES were utilized to collect information from a nationally representative sample of the United States, employing a stratified, multi-stage probability sampling method. Basic information about participants was initially gathered through home interviews, followed by invitations to a Mobile Examination Center (MEC) for a comprehensive examination that included a physical assessment, specialized measurements, and laboratory tests (see http://www.cdc.gov/nchs/nhanes). A nationally representative, non-institutionalized sample of U.S. adults was selected biennially, starting from the 1999-2000 cycle. This study included non-institutionalized U.S. adult participants from seven two-year cycles between 2007 and 2020. The survey protocol was approved by the Ethics Review Board of the National Center for Health Statistics, and all participants gave informed consent, and all participants provided informed consent, agreeing to the use of their data for health statistics research (https://www.cdc.gov/nchs/nhanes/irba98.htm). From the initial sample of 75,402 cases from the NHANES 2007-2020 cycle, 31,400 cases aged <20 years were excluded, and 44,002 adult data were retained as shown in **Figure 1**. By excluding key variables, 14,818 participants were finally included. Weighted analysis showed no significant selection bias was introduced and as shown in **Table 1**).

**Table 1:** Baseline characteristics of participants according to Non-COPD/ COPD from the U.S. National Health and Nutrition Examination Survey.

Parameter	No. of Participants (Weighted %); (mean (SE))			
	All Participants (N = 14, 818)	Non-COPD (N = 14, 189)	COPD (N = 629)	*P-value ^a^*
Life's Simple 7 (LS7)	9.00 (0.04)	9.06 (0.04)	7.67 (0.10)	< 0.001
Life’s Essential 8 (LE8)	72.36 (0.25)	72.66 (0.25)	65.37 (0.72)	< 0.001
Life’s Crucial 9 (LC9)	74.64 (0.23)	74.92 (0.23)	68.07 (0.69)	< 0.001
LC9per-10	7.46 (0.02)	7.49 (0.02)	6.81 (0.07)	< 0.001
Lean Body Mass (LBM)	52.20 (0.15)	52.21 (0.16)	51.84 (0.75)	0.64
Age				
20-44	6851 (47.52)	6776 (49.03)	75 (11.85)	< 0.001
45-64	5160 (37.21)	4853 (36.41)	307 (56.02)	
≥65	2807 (15.27)	2560 (14.56)	247 (32.13)	
Sex				
female	7084 (48.60)	6830 (48.71)	254 (46.10)	0.44
male	7734 (51.40)	7359 (51.29)	375 (53.90)	
Ethnic/race				
white people	6948 (72.08)	6504 (71.50)	444 (85.77)	< 0.001
black people	2850 (9.17)	2768 (9.35)	82 (5.09)	
Mexican people	1995 (7.19)	1968 (7.44)	27 (1.28)	
other people	3025 (11.56)	2949 (11.71)	76 (7.86)	
Marital status				
Married	9021 (65.19)	8640 (65.03)	381 (68.88)	< 0.001
Separated	2936 (18.75)	2889 (19.31)	47 (5.36)	
Never married	2861 (16.07)	2660 (15.66)	201 (25.76)	
Ratio of family income to poverty levels				
<1.3	4173 (18.09)	3960 (17.98)	213 (20.73)	0.26
1.3-3	4523 (27.12)	4334 (27.06)	189 (28.63)	
3-5	3079 (25.03)	2976 (25.20)	103 (20.99)	
≥5	3043 (29.75)	2919 (29.76)	124 (29.65)	
Education levels				
No formal education	2677 (11.43)	2526 (11.24)	151 (16.08)	0.02
Primary school	7435 (56.27)	7144 (56.45)	291 (52.14)	
High school or above	4706 (32.30)	4519 (32.32)	187 (31.77)	
Alcohol consumption status				
former	2000 (11.08)	1848 (10.75)	152 (18.89)	< 0.001
heavy	3174 (21.93)	3070 (22.18)	104 (16.06)	
mild	5403 (39.11)	5163 (39.00)	240 (41.62)	
moderate	2531 (18.92)	2433 (18.94)	98 (18.41)	
never	1710 (8.96)	1675 (9.13)	35 (5.02)	
LS7				
Poor	4164 (24.30)	3864 (23.44)	300 (44.48)	< 0.001
Intermediate,	7252 (50.46)	6957 (50.46)	295 (50.34)	
Ideal	3402 (25.24)	3368 (26.09)	34 (5.18)	
LE8				
Low	839 (4.31)	751 (4.01)	88 (11.42)	< 0.001
Moderate	10029 (65.48)	9544 (64.92)	485 (78.63)	
High	3950 (30.21)	3894 (31.07)	56 (9.95)	
LC9				
Low	481 (2.25)	419 (2.04)	62 (7.21)	< 0.001
Moderate	9685 (62.50)	9198 (61.85)	487 (77.76)	
High	4652 (35.25)	4572 (36.11)	80 (15.03)	
LC9				
Q1	3713 (21.30)	3449 (20.67)	264 (36.13)	< 0.001
Q2	3910 (25.84)	3717 (25.53)	193 (33.13)	
Q3	3456 (24.02)	3337 (24.13)	119 (21.46)	
Q4	3739 (28.83)	3686 (29.67)	53 (9.28)	
LE8/LC9				
HEI	41.10 (0.59)	41.09 (0.59)	41.42 (1.84)	0.85
physical activity	93.70 (0.18)	93.72 (0.18)	93.30 (0.77)	0.59
Smoking	72.52 (0.60)	73.45 (0.60)	50.48 (2.01)	< 0.001
Sleep	84.52 (0.32)	84.73 (0.32)	79.49 (1.52)	< 0.001
BMI	62.43 (0.51)	62.53 (0.52)	60.03 (1.84)	0.20
non-HDL-C	65.38 (0.42)	65.71 (0.41)	57.62 (1.56)	< 0.001
blood glucose	87.88 (0.29)	88.27 (0.30)	78.74 (1.46)	< 0.001
blood pressure	71.70 (0.44)	72.11 (0.45)	62.21 (1.38)	< 0.001
Depression	92.53 (0.22)	92.67 (0.21)	89.35 (1.04)	0.002
LS7				
Blood pressure				
0	2432 (13.61)	2287 (13.41)	145 (18.41)	< 0.001
1	6595 (44.10)	6234 (43.47)	361 (58.83)	
2	5791 (42.29)	5668 (43.12)	123 (22.77)	
Total cholesterol				
0	1821 (12.77)	1722 (12.64)	99 (15.90)	< 0.001
1	6154 (41.12)	5818 (40.41)	336 (57.70)	
2	6843 (46.11)	6649 (46.95)	194 (26.40)	
HbA1c				
0	1118 (5.27)	1044 (5.15)	74 (8.13)	< 0.001
1	4051 (22.32)	3806 (21.66)	245 (37.83)	
2	9649 (72.41)	9339 (73.19)	310 (54.05)	
Smoking				
0	2920 (18.22)	2693 (17.51)	227 (35.10)	< 0.001
1	3608 (25.25)	3332 (24.45)	276 (43.99)	
2	8290 (56.53)	8164 (58.04)	126 (20.91)	
BMI				
0	5355 (34.80)	5130 (34.74)	225 (36.31)	0.16
1	5007 (34.09)	4778 (33.95)	229 (37.52)	
2	4456 (31.11)	4281 (31.32)	175 (26.17)	
Physical activity				
1	2962 (18.77)	2817 (18.69)	145 (20.59)	0.36
2	11856 (81.23)	11372 (81.31)	484 (79.41)	
HEI				
0	7176 (48.33)	6838 (48.18)	338 (51.98)	0.45
1	7125 (47.96)	6851 (48.10)	274 (44.65)	
2	517 (3.71)	500 (3.72)	17 (3.37)	

Percentages were adjusted for NHANES survey weights. ^a^ The P-value was calculated using a chi-square test and Students T test after considering the sampling weights. P-value <0.05.

Data are Mean (standard error) or No. of Participants (Weighted %).

The scoring method for the common indicators of LE8 and LC9 is consistent.

HEI, The dietary practices were assessed at the mobile examination center based on 24-hours dietary recall. For measurement of overall dietary quality used the Healthy Eating Index (HEI) developed by the US Department of Agriculture in 1995, which included the following components: grain, vegetables, fruits, milk, meat, total fat, saturated fat, cholesterol, sodium and food variety.

An overall LS7 score of 0 to 4 was considered poor, 5 to 9 was intermediate, and 10 to 14 was ideal. In brief, the 7 cardiovascular health factors include blood pressure, total cholesterol, glycosylated hemoglobin (HbA1c), smoking, BMI, physical activity, and diet (HEI).

Participants with a LE8 score of 80–100 were considered high CVH; 50–79, moderate CVH; and 0–49 points, low CVH. LE8 scoring algorithm consists of 4 health behaviors (diet, physical activity, nicotine exposure (smoking), and sleep) and 4 health factors (body mass index (BMI), non-high-density-lipoprotein cholesterol (non-HDL-C), blood glucose, and blood pressure).

LC9 scoring algorithm consists of 4 health behaviors (diet (Healthy Eating Index, HEI), physical activity, nicotine exposure (smoking), and sleep duration), 4 health factors (body mass index [(BMI], ), non-high-density-lipoprotein cholesterol (non-HDL-C), blood glucose, and blood pressure) and Mental health (Depression).

At present, there is no recognized and applicable threshold limit for LC9 scores. Therefore, this study presents LC9 levels from multiple dimensions. For example, the following four dimensions: quartile grouping (Q1, Q2, Q3, Q4), grouping based on the LE8 threshold (Low (0–49), Moderate (50–79), High (80–100)), LC9-per10 (continuous variable), LC9 (continuous variable).

### 2.2 Measurement of cardiovascular health score

The ls7 is calculated based on the AHA guidelines for blood pressure, total cholesterol, glycosylated hemoglobin (HbA1c), smoking, BMI, physical activity, and diet (Healthy Eating Index, HEI) (21). The sum of all seven scores is the final ls7 score. Each cardiovascular health factor is categorized into three groups (ideal, moderate, and poor.) A total ls7 score of 0 to 4 is considered poor, 5 to 9 is moderate, and 10 to 14 is ideal (22). The le8 is calculated based on the AHA Guidelines for four health behaviors (diet (Healthy Eating Index, HEI), physical activity, smoking), and sleep and four health factors (BMI, non-high-density lipoprotein cholesterol (non-HDL-C), blood glucose, and blood pressure) (14). Detailed calculations for each indicator have been documented in previous studies (14). Dietary intake was assessed using the Healthy Eating Index (HEI-2015), which is based on data from two 24-hour dietary recalls and food pattern scores provided by the United States Department of Agriculture (USDA). Information on physical activity, medication use, smoking, history of diabetes, and sleep duration was collected using a self-report questionnaire. During the physical examination, weight, blood pressure, and height were measured, and blood pressure was reported as the mean of three measurements. Body mass index was calculated as weight divided by the square of height. Blood samples were analyzed in a central laboratory to assess non-high-density lipoprotein cholesterol (non-HDL-C), glycosylated hemoglobin (HbA1c), and blood glucose. Briefly, each of the 8 CVH indicators was scored on a scale of 0 to 100. The overall le8 score was calculated as the unweighted average of the 8 measures. participants with le8 scores of 80-100 were considered to have high CVH; 50-79, moderate CVH; and 0-49, low CVH. It is reported that consideration of mental health factors is fundamental to achieving optimal and equitable CVH (15). An area of high interest in mental health factors is depression. Depression is an independent non-traditional odds factor for cardiovascular disease (CVD) (23). Depression scores are calculated based on the Patient Health Questionnaire 9 (PHQ-9) score, which is a validated structured questionnaire for depression screening. Higher PHQ-9 scores indicate higher levels of currently present depressive symptoms. Depression scores are designated as 100, 75, 50, 25, and 0, which correspond to 0 to 4, 5 to 9, 10 to 14, 15 to 19, and 20 to 27 in the PHQ-9 score, respectively.19 The lc9 score is calculated as the average of the le8 score and the eight indicators in the depression score (24). The American Cardiovascular Society emphasized the importance of mental health in the prevention of CVD and introduced factors such as depression into a new metric called Life’s Crucial 9 (lc9). Prior to this, only the concept of constructing an lc9 scoring system was proposed, but the American Cardiovascular Society did not formally publish the composition and calculation of the lc9 index (15). The most recent study proposed the process of constructing and calculating the lc9 scoring system (12) and verified that it has a better ability to predict cardiovascular health (12). Specifically, Ge et al. validated the association of lc9 with cardiovascular mortality and all-cause mortality, which improved the cardiovascular health odds scoring system and provided direction for subsequent studies (12). In summary, the addition of depression to the “Life Essential 8 (le8) scale proposed by the American Heart Association to construct the lc9 scoring system to measure CVH has been generally recognized. There is currently no recognized threshold for the lc9 score. Two tests were performed in this study: quartile grouping (Q1, Q2, Q3, Q4) and grouping based on the le8 threshold (Low (0-49), Moderate (50-79), High (80-100)). The scoring method for the common indicators of le8 and lc9 is consistent.

### 2.3 Assessment of LBM

In this study, the assessment of LBM was based on a predictive equation developed by Lee et al. (25), which utilized participant data from the NHANES survey for model construction. A total of 10,518 male and 10,987 female participants in the study underwent dual-energy X-ray bone density (DXA) scans. Multivariate linear regression was used to estimate LBM, with LBM as the dependent variable and predictor variables including age, gender, height (cm), weight (kg), and waist circumference (cm). The linear regression model performed best in terms of consistency [LBM (female: R² = 0.85; male: R² = 0.91)]. The specific LBM formula was: male LBM = 19.363 + 0.001 * age (years) + 0.064 * height (cm) + 0.756 * weight (kg) - 0.366 * waist circumference (cm) - 1.007; female LBM =- 10.683 - 0.039 * Age (years) + 0.186 * Height (cm) + 0.383 * Weight (kg) - 0.043 * Waist (cm) - 0.340 (17).

### 2.4 Definition of COPD

The definition of COPD in this study was based on participants’ self-reported physician diagnosis (26, 27), and COPD was determined by three self-reported questionnaire items: “Has your doctor ever told you that you have chronic bronchitis?”, “Has your doctor ever diagnosed you with emphysema?”, “Is FEV1/FVC <0.7 after inhalation of bronchodilators?”, “Has a doctor or other health professional ever told you that you have COPD?”, “Are you using COPD medications (leukotriene modulators, inhaled corticosteroids, selective phosphodiesterase-4 inhibitors, mast cell stabilizers)?”, Participants who answered “yes” to any of these questions were categorized into the COPD group, while participants who answered “no” to all questions were categorized into the non-COPD group.

### 2.5 Definition of covariates

The Centers for Disease Control and Prevention (CDC) collected demographic characteristics, lifestyle, self-reported health status, physical measurements, and biochemical data on participants through a computer-assisted personal interview system. In this study, demographic information was collected through a questionnaire, which included age (20-44, 45-64, 65 and older, years), gender, Ethnic/race: white people (non-Hispanic white, Hispanics white, Europeans Americans), black people (non-Hispanic black, Indigenous Africans, African Americans), Mexican people, other people, marital status (married, separated, and never married), and household income-to-poverty ratio (less than 1.30, 1.30 to <3.00, 3.00 to <5.00, 5.00 and above, indicates the ratio of family income to the federal poverty level, adjusted for family size, with higher ratios indicating higher income levels), and education level (did not complete high school (less than 11th grade), high school graduation/general education, part of college or more (college graduation and above)). Drinking status was categorized as: current heavy drinkers (≥3 drinks per day for women or ≥4 drinks per day for men or binge drinking on 5 or more days per month); current moderate drinkers (≥2 drinks per day for women or ≥3 drinks per day for men, or binge drinking on 2 or more days per month); current light/moderate drinkers (not falling into the first two categories); ex-drinkers who used to drink but do not now; and no Drinkers.

### 2.6 Statistical analysis

The analysed data were weighted according to NCHS requirements. Participants were divided into two groups based on whether they had COPD or not. Statistical tests for weight adjustment were fully considered. The chi-square test and t-test were applied to examine the demographic characteristics in relation to participants’ COPD status. The association between CVH (ls7, le8, lc9) and COPD was estimated by weighted multivariate logistic regression modeling. The association between the components of CVH (ls7, le8, lc9) and COPD was evaluated using the weighted univariate logistic regression model. P-values, odds ratios (ORs), and 95% confidence intervals (CIs) between CVH and odds of COPD were reported. Three models were developed:(1) Crude model (unadjusted); (2) Model 1, adjusted for age, gender, and race/ethnicity;(3) Model 2, adjusted for age, gender, race/ethnicity, Marital status, education level, Family income-to-poverty ratio, lean body mass and Alcohol consumption status. RCS analyses were used to show linear trends in ls7, le8 and lc9 (entered as a continuous variable into the RCS model) with COPD. The RCS model adjusted for Sex, Age, Ethnic/race, Marital status, Family income-to-poverty ratio, Education levels, lean body mass and Alcohol consumption status. Stratified analyses were conducted for Sex, Age, Ethnic/race, Marital status, Family income-to-poverty ratio, Education levels, lean body mass and Alcohol consumption status using weighted multivariate logistic regression. Additionally, the interaction of lc9 with potential confounders was considered. ROC curve analysis was conducted to assess the predictive ability of ls7, le8 and lc9 for COPD. Results are presented as the area under the ROC curve (AUC) along with the corresponding 95% confidence interval (CI), as well as sensitivity and specificity metrics. The potential mediating of LBM on the relationship between lc9 and COPD odds was estimated using a parallel cross-sectional mediation model implemented. Due to the concurrent measurement of LC9 and LBM, our analysis aimed to explore their statistical relationships rather than establish causal pathways. We further conducted sensitivity analyses using multiple model specifications to assess the robustness of the LC9-COPD association, with covariate adjustments mirroring the primary analytical approach The following sensitivity analyses were conducted: 1. Imputation of variables with missing values in the dataset was performed to test whether the correlation between lc9 and COPD was tested in the complete dataset. 2. After excluding depression and sleep indicators, the correlation between ls7 and COPD was tested. 3. After excluding depression indicators, the correlation between le8 and COPD was tested. All statistical analyses were conducted using R software (version 4.2.2, https://cran.r-project.org/bin/windows/base/old/4.2.2/). Two-sided statistical tests were used, with a significance level set at a *P-value* < 0.05.

## 3 Results

### 3.1 Baseline characteristics

A total of 14, 818 participants were included, and after applying weights, the sample is representative of 99, 106, 357 individuals in the U.S. general population. Among them, 629 were COPD patients, and 14, 189 were non-COPD patients. The proportion of COPD cases (85.77%) among white people is the highest. The COPD group exhibited significantly lower scores in ls7, le8, lc9-per10 and lc9, compared to the non-COPD group (*P-value* < 0.05). Compared with non-COPD participants, significant statistical differences (*P-value* < 0.05) were observed in Age, Ethnic/race, Marital status, Education levels, Alcohol consumption status, and CVH Categorical Variables (ls7, le8, lc9). In comparison with the non-COPD group, the scores of ls7 components (blood glucose, total cholesterol, HbA1c, Smoking), le8 components (Smoking, Sleep, non-HDL-C, blood pressure, blood glucose) and lc9 components (Smoking, Sleep, non-HDL-C, blood glucose, blood pressure, Depression) in the COPD group were lower and as shown in **Table 1**.

### 3.2 Association of LC9 scores with copd odds

After full adjustment for potential confounders, lc9 remained significantly associated with COPD. lc9 scores showed a significant inverse relationship with COPD odds. In Model 2, compared to Q1, the OR (95% CI) and *P for trend* were reported: Q3 (OR = 0.58; 95% CI: 0.42-0.81), Q4 (OR = 0.24; 95% CI: 0.16-0.36)) and *P for trend* < 0.001. Additionally, In Model 2, compared to Low, Moderate (OR = 0.37; 95% CI: 0.23-0.59), High (OR = 0.16; 95% CI: 0.09-0.27) and *P for trend* < 0.001. Each 10-point increase in the lc9 score was associated with a reduced odds of COPD (OR = 0.66; 95% CI: 0.59-0.73) and as shown in **Table 2**. In Model 2, the results of the association between the ls7 component and COPD were as follows: HbA1c (OR = 0.805; 95% CI: 0.681-0.951), Smoking (OR = 0.387; 95% CI: 0.331, 0.452), HEI (OR = 0.774; 95% CI: 0.630-0.951 and as shown in **Supplementary Table 4**. In Model 2, the results of the association between the le8/lc9 component and COPD were as follows: Smoking (OR = 0.984; 95% CI: 0.981-0.987), Sleep (OR = 0.387; 95% CI: 0.331- 0.452), blood glucose (OR = 0.993; 95% CI: 0.988-0.998), Depression (OR = 0.992; 95% CI: 0.987-0.997) and as shown in **Supplementary Table 5**.

**Table 2:** Association of LC9 Scores with COPD Risk.

Parameter	Crude model		Model 1		Model 2		*P for trend*
	OR (95%CI)	*P-value*	OR (95%CI)	*P-value*	OR (95%CI)	*P-value*	
Per 10-score increase	0.63 (0.58, 0.68)	< 0.001	0.65 (0.60, 0.71)	< 0.001	0.66 (0.59, 0.73)	< 0.001	–
Low	ref	ref	ref	ref	ref	ref	< 0.001
Moderate	0.35 (0.24, 0.53)	< 0.001	0.32 (0.21, 0.50)	< 0.001	0.37 (0.23, 0.59)	< 0.001	
High	0.12 (0.07, 0.19)	< 0.001	0.13 (0.08, 0.22)	< 0.001	0.16 (0.09, 0.27)	< 0.001	
Q1	ref	ref	ref	ref	ref	ref	< 0.001
Q2	0.74 (0.56, 0.98)	0.03	0.75 (0.56, 1.00)	0.05	0.80 (0.59, 1.07)	0.13	
Q3	0.51 (0.38, 0.69)	< 0.001	0.55 (0.41, 0.75)	<0.001	0.58 (0.42, 0.81)	0.002	
Q4	0.18 (0.12, 0.27)	< 0.001	0.22 (0.15, 0.33)	< 0.001	0.24 (0.16, 0.36)	< 0.001	

OR odds ratio, CI confdence interval.

Crude model, No adjustment for any potential influence factors.

Model 1, Adjusted for Sex, Age and Ethnic/race.

Model 2, Adjusted for Sex, Age, Ethnic/race, Marital status, Family income-to-poverty ratio, Education levels, lean body mass and Alcohol consumption status.

LC9 scoring algorithm consists of 4 health behaviors (diet (HEI), physical activity, nicotine exposure (smoking), and sleep) and 4 health factors (body mass index (BMI), non-high-density-lipoprotein cholesterol (Non-HDL-c), blood glucose, and blood pressure) and Depression. At present, there is no recognized and applicable threshold limit for LC9 scores. Therefore, this study presents LC9 levels from multiple dimensions. For example, the following four dimensions: quartile grouping (Q1, Q2, Q3, Q4), grouping based on the LE8 threshold (Low (0–49), Moderate (50–79), High (80–100)), LC9-per10 (continuous variable), LC9 (continuous variable).

### 3.3 RCS analysis

**Figure 2:**
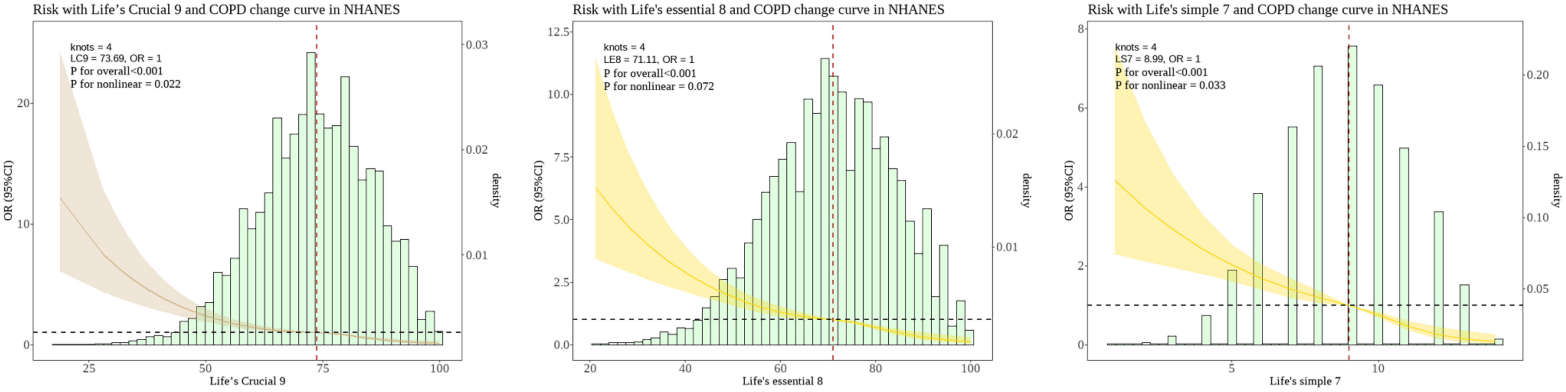
Nonlinear associations between the LC9 LE8 and LS7 scoring systems and the risk of COPD in the NHANES. The dashed line represents the threshold where the OR=1. The shaded area represents the 95% confidence interval. The histogram illustrates the population distribution of "LC9" "LE8" and "LS7" scores. LC9, Life's Crucial 9; LE8, Life's Essential 8; LS7, Life’s Simple 7; OR, Odds Ratio.

The RCS analysis revealed that lc9 had nonlinear relationship with COPD (*P for nonlinear* = 0.022). To validate the association of lc9 with COPD, we carried out two tests: 1. le8 (excluding the depression indicator) was related to COPD, but no significant non-linear relationship was identified. 2. ls7 (excluding the depression and sleep indicators) was related to COPD and a non-linear relationship. The RCS analysis revealed that ls7 had nonlinear relationship with COPD (*P for nonlinear* = 0.033) and as shown in **Figure 2**.

### 3.4 LC9 with COPD odds subgroup analysis

Overall trend: The lc9 scores of quartile grouping and threshold grouping based on le8 were correlated with a decreased odds of COPD. The stratified analysis demonstrated that in all subgroups, a higher lc9 score was significantly and negatively associated with the reduced odds of COPD. The main findings encompassed: gender, age, ethnicity, marital status, and educational level. The lc9 high and Q4 groups consistently showed the strongest associations. A significant interaction was observed between lc9 and ethnicity/race (*P for interaction* = 0.02) and as shown in **Table 3**.

**Table 3:** Association of LC9 Scores with COPD Risk: Subgroup Analysis.

Grouping based on the LE8 threshold (Low (0–49), Moderate (50–79), High (80–100))								Quartile grouping (Q1, Q2, Q3, Q4)								
Parameter	Low	Moderate		High		*P for trend*	*P for interaction*	Q1	Q2		Q3		Q4		*P for trend*	*P for interaction*
		OR (95%CI)	*P-value*	OR (95%CI)	*P-value*				OR (95%CI)	*P-value*	OR (95%CI)	*P-value*	OR (95%CI)	*P-value*		
Sex																
Female	ref	0.34(0.19, 0.63)	< 0.001	0.11(0.05, 0.23)	< 0.001	< 0.001	0.41	ref	0.86(0.61, 1.21)	0.38	0.76(0.54, 1.07)	0.12	0.20(0.11, 0.36)	< 0.001	< 0.001	0.12
Male	ref	0.40(0.20, 0.84)	0.02	0.20(0.09, 0.49)	< 0.001	< 0.001		ref	0.73(0.46,1.14)	0.17	0.41(0.24,0.71)	0.002	0.24(0.14,0.44)	< 0.001	< 0.001	
Age																
20-44	ref	0.71(0.23,2.13)	0.53	0.19(0.04,0.88)	0.03	0.001	0.11	ref	0.48(0.23,1.03)	0.06	0.43(0.17,1.07)	0.07	0.08(0.02,0.32)	< 0.001	< 0.001	0.06
45-64	ref	0.35(0.20,0.60)	< 0.001	0.13(0.05,0.29)	< 0.001	< 0.001		ref	0.90(0.61,1.34)	0.61	0.60(0.39,0.94)	0.03	0.20(0.11,0.35)	< 0.001	< 0.001	
≥65	ref	0.38(0.16,0.93)	0.03	0.26(0.10,0.67)	0.01	0.02		ref	0.79(0.51,1.22)	0.28	0.63(0.38,1.03)	0.07	0.50(0.27,0.91)	0.02	0.02	
Ethnic/race																
white people	ref	0.38(0.21,0.69)	0.002	0.18(0.09,0.34)	< 0.001	< 0.001	0.02	ref	0.76(0.54,1.06)	0.11	0.64(0.45,0.92)	0.02	0.26(0.17,0.41)	< 0.001	< 0.001	0.02
black people	ref	0.34(0.15, 0.76)	0.01	0.18(0.06, 0.52)	0.002	0.01		ref	0.82(0.49, 1.39)	0.46	0.32(0.16, 0.67)	0.003	0.41(0.17, 0.98)	0.05	0.001	
Mexican people	ref	0.66(0.09, 4.73)	0.68	0.19(0.02, 2.04)	0.17	0.03		ref	0.51(0.18, 1.45)	0.20	0.25(0.08, 0.81)	0.02	0.09(0.01, 1.05)	0.051	0.01	
other people	ref	0.25(0.10, 0.67)	0.01	0.02(0.01, 0.08)	< 0.001	< 0.001		ref	1.42(0.51, 3.95)	0.50	0.25(0.07, 0.94)	0.04	0.07(0.02, 0.28)	< 0.001	< 0.001	
Marital status																
Married	ref	0.54(0.26, 1.09)	0.08	0.25(0.11, 0.54)	< 0.001	< 0.001	0.07	ref	0.91(0.62, 1.33)	0.61	0.69(0.48, 0.99)	0.04	0.28(0.18, 0.45)	< 0.001	< 0.001	0.18
Separated	ref	0.34(0.11, 1.07)	0.06	0.05(0.01, 0.30)	0.002	< 0.001		ref	1.13(0.43, 2.99)	0.80	0.50(0.10, 2.52)	0.40	0.16(0.03, 0.96)	0.05	0.04	
Never married	ref	0.26(0.13,0.53)	< 0.001	0.09(0.03,0.23)	< 0.001	< 0.001		ref	0.56(0.34,0.92)	0.02	0.37(0.18,0.74)	0.01	0.15(0.07,0.33)	< 0.001	< 0.001	
Education levels																
No formal education	ref	0.24(0.11,0.54)	< 0.001	0.06(0.02,0.18)	< 0.001	< 0.001	0.58	ref	0.60(0.36,0.98)	0.04	0.43(0.21,0.89)	0.02	0.11(0.02,0.52)	0.01	< 0.001	0.12
Primary school	ref	0.44(0.20, 0.95)	0.04	0.22(0.09, 0.52)	< 0.001	< 0.001		ref	1.13(0.72, 1.77)	0.58	0.91(0.52, 1.58)	0.74	0.32(0.18, 0.58)	< 0.001	< 0.001	
High school or above	ref	0.41(0.21, 0.80)	0.01	0.15(0.06, 0.36)	< 0.001	< 0.001		ref	0.57(0.35, 0.95)	0.03	0.36(0.20, 0.66)	0.001	0.24(0.11, 0.49)	< 0.001	< 0.001	
Ratio of family income to poverty levels																
< 1.3	ref	0.39(0.21,0.72)	0.003	0.11(0.03,0.37)	< 0.001	< 0.001	0.39	ref	0.51(0.35,0.74)	< 0.001	0.32(0.16,0.63)	0.001	0.19(0.06,0.63)	0.01	< 0.001	0.06
1.3-3	ref	0.28(0.12, 0.61)	0.002	0.13(0.05, 0.38)	< 0.001	0.003		ref	0.77(0.44, 1.36)	0.37	0.63(0.33, 1.23)	0.17	0.36(0.16, 0.85)	0.02	0.02	
3-5	ref	1.36(0.34, 5.43)	0.66	0.41(0.10, 1.75)	0.23	< 0.001		ref	1.32(0.69, 2.52)	0.39	0.47(0.20, 1.11)	0.08	0.15(0.06, 0.38)	< 0.001	< 0.001	
≥5	ref	0.19(0.03, 1.18)	0.07	0.11(0.02, 0.71)	0.02	0.01		ref	0.82(0.43, 1.59)	0.56	0.91(0.46, 1.80)	0.78	0.33(0.16, 0.67)	0.003	0.003	
Alcohol consumption status																
former	ref	0.73(0.35, 1.51)	0.39	0.38(0.12, 1.18)	0.09	0.06	0.37	ref	0.80(0.42, 1.53)	0.49	0.65(0.29, 1.45)	0.29	0.26(0.08, 0.80)	0.02	0.02	0.09
heavy	ref	0.18(0.03, 1.14)	0.07	0.04(0.01, 0.29)	0.002	< 0.001		ref	0.69(0.24, 1.94)	0.47	0.04(0.01, 0.24)	< 0.001	0.11(0.02, 0.53)	0.01	< 0.001	
mild	ref	0.28(0.12, 0.65)	0.004	0.09(0.04, 0.23)	< 0.001	< 0.001		ref	0.85(0.56, 1.28)	0.42	0.57(0.37, 0.88)	0.01	0.21(0.12, 0.38)	< 0.001	< 0.001	
moderate	ref	0.50(0.17, 1.49)	0.21	0.38(0.09, 1.69)	0.20	0.38		ref	1.18(0.55, 2.51)	0.67	1.29(0.63, 2.62)	0.48	0.67(0.28, 1.59)	0.35	0.45	
never	ref	0.29(0.13, 0.63)	0.002	0.11(0.03, 0.39)	< 0.001	0.002		ref	0.50(0.29,0.85)	0.01	0.43(0.21,0.86)	0.02	0.11(0.03,0.38)	< 0.001	< 0.001	

Model adjusted for Sex, Age, Ethnic/race, Marital status, Family income-to-poverty ratio, Education levels, lean body mass and Alcohol consumption status.

OR odds ratio, CI confdence interval.

LC9 scoring algorithm consists of 4 health behaviors (diet (HEI), physical activity, nicotine exposure (smoking), and sleep) and 4 health factors (body mass index (BMI), non-high-density-lipoprotein cholesterol (Non-HDL-c), blood glucose, and blood pressure) and Depression. At present, there is no recognized and applicable threshold limit for LC9 scores. Therefore, this study presents LC9 levels from multiple dimensions. For example, quartile grouping (Q1, Q2, Q3, Q4) and grouping based on the LE8 threshold (Low (0–49), Moderate (50–79), High (80–100)).

### 3.5 ROC analysis of LC9 in predicting COPD

**Figure 3:**
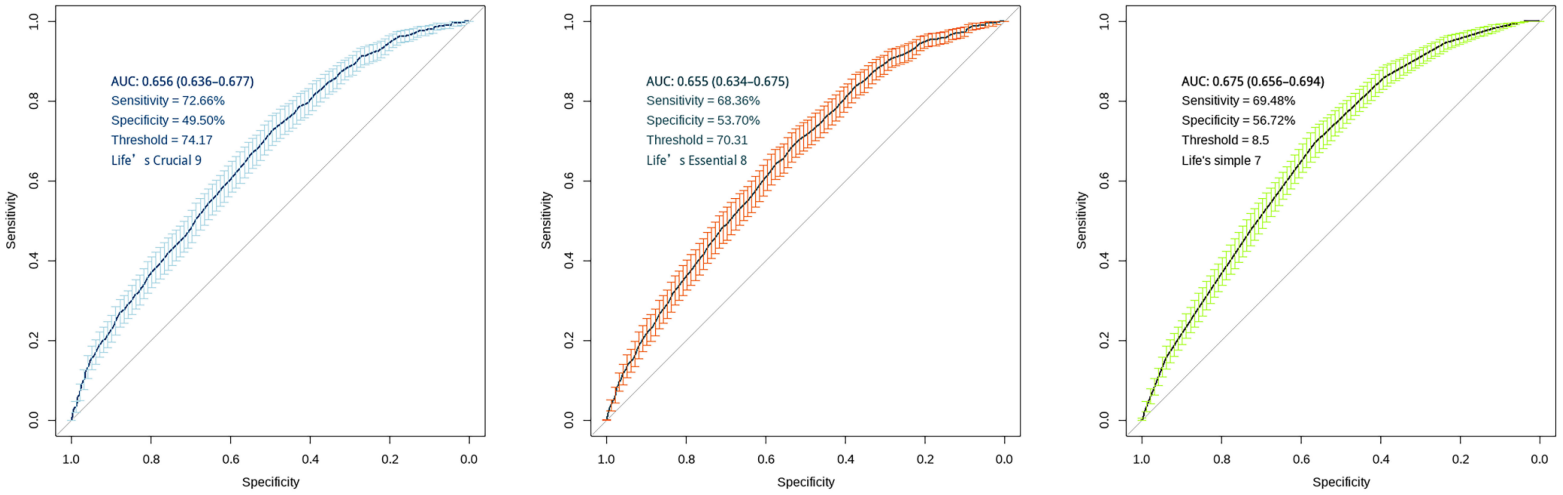
ROC curves for "LC9", "LE8" and "LS7" scoring systems. The figure displays AUC with 95% confidence intervals, sensitivity, specificity, and optimal threshold values. The diagonal line in both panels represents the line of no-discrimination (AUC = 0.5). Error bars indicate the confidence intervals at various points along the curve.

ROC curves were analyzed for the efficacy of ls7, le8 and lc9 in predicting COPD odds. To validate the association of lc9 predicts the odds of COPD, we carried out two tests: 1. le8 (excluding the depression indicator) predicts the odds of COPD. 2. ls7 (excluding the depression and sleep indicators) predicts the odds of COPD. The AUC for lc9 score is 0.656 (0.636-0.677), with an optimal threshold of 74.17, sensitivity of 72.66%, and specificity of 49.50%. The AUC for le8 score is 0.655 (0.634-0.675), with an optimal threshold of 70.31, sensitivity of 68.36%, and specificity of 53.70%. The AUC for ls7 score is 0.675 (0.656-0.694), with an optimal threshold of 8.5, sensitivity of 69.48%, and specificity of 56.72% and as shown in **Figure 3**.

### 3.6 Cross-sectional mediation model

**Figure 4:**
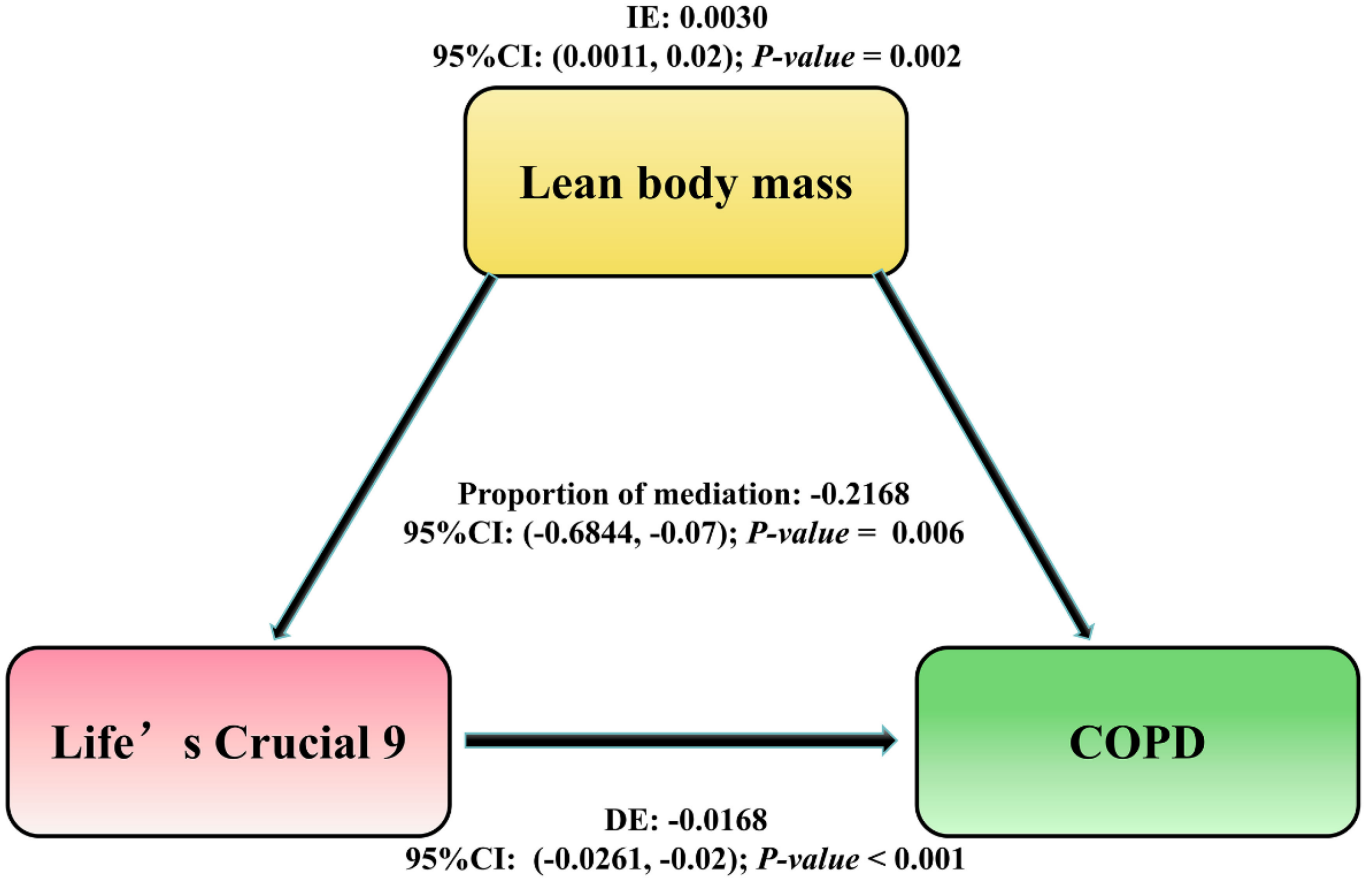
The mediation pathway analysis of the association between LC9 score and the risk of COPD through LBM. The arrows indicate the direction of relationships between the variables. DE, Direct Effect (the direct relationship between LC9 and COPD); IE, Indirect Effect (the influence on COPD mediated through LBM); Proportion of mediation = IE / (DE + IE); OR, Odds Ratio.

The proportion of mediation was OR = -0.2168; 95% CI: -0.6844–0.07; *P-value* = 0.006). These findings suggest that LBM plays a suggestive mediating potential in the odds of COPD by lc9 score and as shown in **Figure 4**.

### 3.7 Sensitivity analysis

To verify the stability of the association between lc9 and COPD, the following three tests were carried out: 1. Data imputation methods were employed to impute the variables with missing values. The association between lc9 and COPD was tested in the complete dataset. 2. The association between le8 (excluding the depression indicator) and COPD was examined. 3. The association between ls7 (excluding the depression and sleep indicators) and COPD was investigated. The results demonstrated that all CVH indicators (ls7, le8, lc9) were correlated with COPD. The results of the study are reported in **Supplementary Tables 1**–**3**.

## 4 Discussion

This study is a continuous cross-sectional study based on 7 cycles of NHANES data 2007-2020. There were 3 important findings. First, the lc9 score was negatively associated with the odds of COPD, and LBM had a suggestive mediating potential. Second, compared to the non-COPD group, the COPD group had lower levels of variables including health behaviors healthy (smoking, Sleep), health factors (non-HDL-C, blood glucose, blood pressure) and mental health (Depression). Additionally, White people had the highest percentage in the COPD group. Additionally, lc9 had interaction with race and was more protective for White people.

We observed that the levels of Smoking, Sleep, non-HDL-C, blood glucose, blood pressure, and Depression in the COPD group at baseline were lower than those in the normal group. Furthermore, white people accounted for the highest proportion within the COPD group. Prior studies have examined the correlation between le8 and COPD, noting that compared with the non-COPD group, the levels of physical activity, smoking, sleep health, blood lipids, blood glucose, and blood pressure in the COPD group were significantly lower (28). Moreover, the COPD group with higher levels of smoking and depression exhibited a higher odds of mortality (29). Additionally, a 3-month cohort study discovered that among COPD patients, the majority were male (94%) and white (91%), with a relatively higher proportion of white COPD patients (30, 31). These findings are analogous to ours. lc9 is a measure of CVH (12). The association between COPD and CVH has been well documented (26, 28). The possible reasons for the high proportion of white participants in the NHANES study are shown below. NHANES utilizes a complex stratified multistage probability cluster sampling design, which, although designed to ensure representativeness, may result in a higher proportion of white participants if certain minority neighborhoods are underrepresented in selected clusters or strata (32). Second, it is more difficult for low-income groups to coordinate time to participate in on-site inspections. Further, such as language barriers, cultural differences, and distrust of medical research. These factors may contribute to lower participation rates among minorities. A similar imbalance in racial distribution was found (33).

Our results that lc9 was negatively associated with the odds of COPD, and this association was consistent across the overall sample, subgroup analyses, and sensitivity analyses. From the perspective of epidemiologic studies, Liu et al. concluded that CVH is negatively associated with the odds of developing COPD (28), and that maintaining an optimal CVH level is beneficial in stopping the development of COPD (28). In a population ≥40 years old, the higher the le8 score the lower the odds of COPD (26). Additionally, ls7 scores have been associated with lung function as well as the prevalence of COPD (34).The lc9 score consists of four health behaviors (diet, physical activity, nicotine exposure, and sleep), four health factors (BMI, non-HDL-C, blood glucose and blood pressure), and mental health (depression) (12).The lc9 considers the roles of sleep and mental health in COPD and may capture overlapping odds factors for COPD development.

Specifically, the lc9 component has been associated with a reduced incidence of COPD. In terms of diet, higher diet quality (e.g., adherence to a Mediterranean dietary pattern) reduces COPD odds. However, adherence to Western dietary patterns, e.g., high amounts of meat or processed meats, saturated fatty acids, increases odds (35). Genetic evidence points that exercise promotes the differentiation of lung tissue stem cells, remodeling blood vessel formation and enhancing lung ventilation (36). Additionally, smoking is the most important environmental odds factor for COPD. Chronic inflammation induced by smoking will directly contribute to COPD by reducing insulin action and elevating blood glucose levels, leading to decreased lung function (37). Additionally, sleep deprivation is associated with mildly reduced FVC (-5%) and FEV 1 (-6%) (38). It has been noted that poor sleep quality are significantly associated with the severe COPD (39). Low LBM is associated with accelerated lung function decline in COPD patients, while the opposite is true for high LBM. (40). Abdominal obesity accumulates large amounts of visceral fat and increases the odds of COPD (41). The excess visceral fat is an excellent pro-inflammatory mediator that attracts inflammatory cells and amplifies the inflammatory process, leading to alterations in the structure of the small airways (42). Mechanistic studies have shown that lipid molecules and their metabolic processes may contribute to COPD development by increasing inflammatory substances (43). Additionally, oxidative stress in which hyperlipidemia induces mitochondrial damage produces excess reactive oxygen species that impair lung function (44). Patients with type 2 diabetes have been reported to be more likely to develop COPD (45). A retrospective study showed that dyslipidemia, fasting hyperglycemia, abdominal obesity, and hypertension, were independently associated with impaired lung function (46). Epidemiologic and genetic evidence agree that depression may play an important role in the prevalence of COPD, clearly indicating that depression may be an etiologic factor in COPD (47). In addition, pro-inflammatory cytokines (e.g., IL-6 and c-reactive protein) may play a role in the relationship between depressive symptoms and lung function in older adults, causing endothelial dysfunction and reduced alveolar function (48), promoting the development of COPD. In summary, the association of lc9 with COPD may reflect the role of systemic inflammation a common pathway linking cardiovascular and pulmonary pathologies. The elevated levels of C-reactive protein and IL-6 (both of which are associated with poor CVH indices) may promote alveolar destruction and airway remodeling through activation of matrix metalloproteinases (49). Our results extend the predictive value of lc9 from cardiovascular outcomes to the domain of COPD odds, suggesting that lc9 may play a dual role in COPD prevention. The ability of lc9 to reduce the odds of COPD was stronger than that of the lc9 component alone.

Our findings suggest that lc9 may alleviate COPD odds by improving LBM. cross-sectional pathway analysis indicated a potential mediating. LBM is a key indicator of muscle mass and metabolic health (50). LBM is associated with CVH, lung function and respiratory health (51, 52). Improving lifestyle and increasing LBM (53) can reduce COPD odds. Furthermore, epidemiologic and genetic evidence agree on a negative association between LBM and COPD and also support a unidirectional causal relationship (54). This finding suggests that increasing LBM may provide additional benefits for the prevention and management of COPD beyond traditional dietary and exercise interventions.

We found a nonlinear relationship between lc9 score and COPD odds. In other words, the OR of the lc9 score associated with COPD was significantly lower in the lower range of the corresponding score and subsequently stabilized at higher values. Although previous studies have shown a positive linear relationship between LS 7 scores and lung function (55), LE 8 showed a nonlinear negative correlation with spirometry or COPD (28). Furthermore, a NHANES study noted a linear negative association between LE 8 and odds of COPD (26). The inconsistent results may be due to the different age composition of the populations studied. When the study population was at ≥40 years old, CVH was linearly associated with the possibility of COPD. The reason is that inflammatory response is more pronounced in middle-aged and older groups, which may lead to a more direct and linear relationship between CVH indicators and disease odds (56). ROC results show low specificity and may be hindered in predicting disease. The findings suggest that LBM is an important node in the path between lc9 and COPD odds. Low-specificity models may produce a higher proportion of false-positive predictions in populations with lower disease prevalence. This may result in a discrepancy between the actual prevalence rate and the false positives that are misclassified by the model. This may be linked to inadequate subgroup sample sizes and limited inclusion of covariates.

The findings suggest a significant interaction of lc9 with race, with stronger protective associations for whites. Earlier uptake of smoking cessation interventions may result in greater gains for whites. Because higher smoking rates in other racial groups amplify the association of “nicotine exposure” indicators (e.g., smoking cessation) with protection in lc9. (57). African-American populations live in areas with higher mean annual PM2.5 concentrations than white people (57, 58), and PM2.5 is able to penetrate deep lung tissues, triggering oxidative stress and inflammatory responses that lead to mitochondrial dysfunction and lung injury (59), contributing to acute exacerbations of COPD (60). The anti-inflammatory capacity of lc9 metabolic indicators (e.g., BMI, glycemic control) may be weakened. Additionally, the assessment of depression in the lc9 (PHQ-9 scale) may underestimate the mental health burden of minorities, who are more likely to attribute psychological problems to physiological or social factors than to direct mood disorders (61). Hispanic immigrants (especially Mexicans), who are overrepresented in NHANES, may have systematically higher lc9 scores due to pre-immigrant health behavioral strengths (e.g., low processed food intake, high physical activity). This “initial health advantage” may mask the ability of lc9 scores to reduce the odds of COPD (62). This possible explanation helps to explain our findings.

This study has several strengths. First, it is a study based on coverage of different age, race, gender, and socioeconomic groups in the U.S. through stratified multistage probability sampling, and the results are generalizable to the entire country. Second, all laboratory tests were performed through a standardized process certified by the CDC, ensuring comparable and reliable data. Moreover, the study extends the predictive value of lc9 from cardiovascular outcomes to the COPD odds domain, bringing together standardized health indicators (lc9) that may play a dual role in COPD prevention. However, some limitations should not be ignored. First, this was a cross-sectional study and no causal association between lc9 and COPD could be inferred, and reverse causality cannot be ruled out in observational studies. Second, this study was based on a questionnaire, which may be subject to recall bias or social desirability bias. Some biomarkers (e.g., glucose, lipids) were based on single measurements only, which may not reflect long-term exposure levels. Additionally, the small sample sizes of some subgroups resulted in wide confidence intervals for the estimates, and the results need to be interpreted with caution. Further, NHANES excluded hospitalized patients and specific institutionalized populations, which may be subject to Newman’s bias and may lead to overrepresentation of healthy populations and underestimation of COPD odds. Moreover, this study relied on questionnaires and lacked sufficient follow-up data, thus limiting in-depth exploration of the association between lc9 and COPD. Importantly, despite adjusting for demographic and socioeconomic variables, unmeasured confounders (use of biomass fuels, air pollution, occupational history, genetic susceptibility, household pollution, environmental factors such as place of residence/zip code/geographic area, etc.) may influence the association between lc9 and COPD. Moreover, we did not consider associations with healthy migration in the NHANES study. Then, the specificity of the model in this study (49.50%, 53.70%, 56.72%) suggests that its ability to distinguish true-negative cases is limited, which may lead to an elevated false-positive rate. Finally, as the mediating potential accounted for a relatively small proportion, there may have been more important mediating potential factors explored. Notably, the results are difficult to generalize to other states and countries. The cross-sectional design precludes establishing temporal prioritization between LC9 and LBM. Although our findings are consistent with the hypothesis of experimental studies suggesting independent roles for LC9 and LBM in lung function, future longitudinal or interventional studies are needed to unravel their causal interactions.

In conclusion, our results suggest that the higher the lc9 score, the lower the odds of COPD, and that LBM plays an important mediating potential. Optimizing lifestyle factors, particularly enhancing LBM, may contribute to mitigating COPD odds. Based on cross-sectional study, the lc9 scoring system could serve as a tool to identify odds associations.

## Data Availability

The datasets presented in this study can be found in online repositories. The names of the repository/repositories and accession number(s) can be found below: http://www.cdc.gov/nchs/nhanes.htm.
