# Supplementary material for: Association of cardiovascular health with COPD (NHANES 2007-2020): mediating potential of lean body mass
Source: Front Endocrinol (Lausanne). 2025 Apr 4;16:1539550. doi: 10.3389/fendo.2025.1539550 (PMC12006009; doi:10.3389/fendo.2025.1539550)
Supplement: Supplementary file 1 [file DataSheet1.docx]

| **Supplementary Table 1\|** Association of LC9 Scores with COPD Risk. (**Dataset imputation**). | | | | | | |  |
| --- | --- | --- | --- | --- | --- | --- | --- |
| **Parameter** | **Crude model** | | **Model 1** | | **Model 2** | | ***P for trend*** |
|  | **OR (95%CI)** | ***P-value*** | **OR (95%CI)** | ***P-value*** | **OR (95%CI)** | ***P-value*** |  |
| Per 10-­score increase | 0.70 (0.66,0.73) | < 0.001 | 0.73 (0.69,0.77) | < 0.001 | 0.75 (0.71, 0.79) | < 0.001 | - |
| Low | ref | ref | ref | ref | ref | ref | **< 0.001** |
| Moderate | 0.49 (0.42,0.56) | < 0.001 | 0.51 (0.44,0.60) | < 0.001 | 0.55 (0.47, 0.63) | < 0.001 |  |
| High | 0.17 (0.13,0.22) | < 0.001 | 0.22 (0.17,0.29) | < 0.001 | 0.25 (0.19, 0.33) | < 0.001 |  |
| Q1 | ref | ref | ref | ref | ref | ref | **< 0.001** |
| Q2 | 0.60 (0.50,0.72) | < 0.001 | 0.62 (0.52,0.74) | < 0.001 | 0.65 (0.54, 0.78) | < 0.001 |  |
| Q3 | 0.47 (0.38,0.58) | < 0.001 | 0.52 (0.42,0.65) | < 0.001 | 0.57 (0.45, 0.71) | < 0.001 |  |
| Q4 | 0.25 (0.19,0.31) | < 0.001 | 0.31 (0.25,0.40) | < 0.001 | 0.35 (0.28, 0.45) | < 0.001 |  |
| OR odds ratio, CI confdence interval. | | | | | | | |
| Crude model, No adjustment for any potential influence factors. | | | | | | | |
| Model 1, Adjusted for Sex, Age and Ethnic/race. | | | | | | | |
| Model 2, Adjusted for Sex, Age, Ethnic/race, Marital status, Family income-to-poverty ratio Education levels, lean body mass and Alcohol consumption status. | | | | | | | |
| LC9 scoring algorithm consists of 4 health behaviors (diet (HEI), physical activity, nicotine exposure (smoking), and sleep) and 4 health factors (body mass index (BMI), non-high-density-lipoprotein cholesterol (Non-HDL-c), blood glucose, and blood pressure) and Depression. At present, there is no recognized and applicable threshold limit for LC9 scores. Therefore, this study presents LC9 levels from multiple dimensions. For example, the following four dimensions: quartile grouping (Q1, Q2, Q3, Q4), grouping based on the LE8 threshold (Low (0–49), Moderate (50–79), High (80–100)), LC9-per10 (continuous variable), LC9 (continuous variable). | | | | | | | |

| **Supplementary Table 2\|** Association of LS7 Scores with COPD Risk. | | | | | | |  |
| --- | --- | --- | --- | --- | --- | --- | --- |
| **Parameter** | **Crude model** | | **Model 1** | | **Model 2** | | ***P for trend*** |
|  | **OR (95%CI)** | ***P-value*** | **OR (95%CI)** | ***P-value*** | **OR (95%CI)** | ***P-value*** |  |
| Poor | ref | ref | ref | ref | ref | ref | **< 0.001** |
| Intermediate, | 0.53 (0.41, 0.67) | < 0.001 | 0.62 (0.49, 0.80) | <0.001 | 0.64 (0.49, 0.83) | 0.001 |  |
| Ideal | 0.10 (0.07, 0.17) | < 0.001 | 0.18 (0.11, 0.29) | < 0.001 | 0.19 (0.11, 0.32) | < 0.001 |  |
| OR odds ratio, CI confdence interval. | | | | | | | |
| Crude model, No adjustment for any potential influence factors. | | | | | | | |
| Model 1, Adjusted for Sex, Age and Ethnic/race. | | | | | | | |
| Model 2, Adjusted for Sex, Age, Ethnic/race, Marital status, Family income-to-poverty ratio Education levels, lean body mass and Alcohol consumption status. | | | | | | | |
| An overall LS7 score of 0 to 4 was considered poor, 5 to 9 was intermediate, and 10 to 14 was ideal. In brief, the 7 cardiovascular health factors include LS7 Score BP, LS7 Score TC, LS7 Score HbA1c, LS7 Score Smoke, LS7 Score BMI, LS7 Score PA, LS7 Score HEI. | | | | | | | |

| **Supplementary Table 3\|** Association of LE8 Scores with COPD Risk. | | | | | | |
| --- | --- | --- | --- | --- | --- | --- |
| **Parameter** | **Crude model** | | **Model 1** | | **Model 2** | |
|  | **OR (95%CI)** | ***P-value*** | **OR (95%CI)** | ***P-value*** | **OR (95%CI)** | ***P-value*** |
| Low | ref | ref | ref | ref | ref | ref |
| Moderate | 0.43(0.30,0.60) | < 0.001 | 0.43(0.30, 0.62) | < 0.001 | 0.46(0.32,0.66) | < 0.001 |
| High | 0.11(0.07,0.18) | < 0.001 | 0.14(0.09, 0.22) | < 0.001 | 0.16(0.10,0.25) | < 0.001 |
| OR odds ratio, CI confdence interval. | | | | | | |
| Crude model, No adjustment for any potential influence factors. | | | | | | |
| Model 1, Adjusted for Sex, Age and Ethnic/race. | | | | | | |
| Model 2, Adjusted for Sex, Age, Ethnic/race, Marital status, Family income-to-poverty ratio Education levels, lean body mass and Alcohol consumption status. | | | | | | |
| Participants with a LE8 score of 80–100 were considered high CVH; 50–79, moderate CVH; and 0–49 points, low CVH. LE8 scoring algorithm consists of 4 health behaviors (diet, physical activity, nicotine exposure, and sleep duration) and 4 health factors (body mass index [BMI], non-high-density-lipoprotein cholesterol, blood glucose, and blood pressure). | | | | | | |

| **Supplementary Table 4\|** Association of components of LS7 Scores with COPD Risk. | | | | | | |
| --- | --- | --- | --- | --- | --- | --- |
| **Parameter** | **Crude model** | | **Model 1** | | **Model 2** | |
|  | **OR (95%CI)** | ***P-value*** | **OR (95%CI)** | ***P-value*** | **OR (95%CI)** | ***P-value*** |
| Blood pressure | 0.603 (0.528, 0.689) | < 0.001 | 0.873 (0.738, 1.031) | 0.109 | 0.911 (0.769, 1.080) | 0.277 |
| Total cholesterol | 0.626 (0.548, 0.714) | < 0.001 | 0.863 (0.730, 1.020) | 0.083 | 0.879 (0.747, 1.034) | 0.118 |
| HbA1C | 0.576 (0.501, 0.662) | < 0.001 | 0.777 (0.654, 0.924) | 0.005 | 0.805 (0.681, 0.951) | 0.012 |
| Smoking | 0.447 (0.399, 0.499) | < 0.001 | 0.385 (0.333, 0.445) | < 0.001 | 0.387 (0.331, 0.452) | < 0.001 |
| BMI | 0.902 (0.786, 1.036) | 0.143 | 0.927 (0.798, 1.076) | 0.316 | 0.907 (0.732, 1.124) | 0.369 |
| Physical activity | 0.887 (0.684, 1.149) | 0.36 | 1.019 (0.777, 1.336) | 0.891 | 1.035 (0.788, 1.359) | 0.804 |
| HEI | 0.877 (0.714, 1.077) | 0.209 | 0.707 (0.574, 0.872) | 0.001 | 0.774 (0.630, 0.951) | 0.015 |
| OR odds ratio, CI confdence interval. | | | | | | |
| Crude model, No adjustment for any potential influence factors. | | | | | | |
| Model 1, Adjusted for Sex, Age and Ethnic/race. | | | | | | |
| Model 2, Adjusted for Sex, Age, Ethnic/race, Marital status, Family income-to-poverty ratio Education levels, lean body mass and Alcohol consumption status. | | | | | | |
| In brief, the 7 cardiovascular health factors include blood pressure, total cholesterol, glycosylated hemoglobin (HbA1c), smoking, BMI, physical activity (PA), and diet (Healthy Eating Index, HEI). | | | | | | |

| **Supplementary Table 5\|** Association of components of L C9 and LE8 with COPD. | | | | | | |
| --- | --- | --- | --- | --- | --- | --- |
| **Parameter** | **Crude model** | | **Model 1** | | **Model 2** | |
|  | **OR (95%CI)** | ***P-value*** | **OR (95%CI)** | ***P-value*** | **OR (95%CI)** | ***P-value*** |
| HEI | 1.000 (0.997, 1.004) | *0.850* | 0.996 (0.993, 1.000) | *0.032* | 0.998 (0.995, 1.001) | *0.252* |
| physical activity | 0.998 (0.993, 1.004) | 0.579 | 1.002 (0.996, 1.008) | 0.468 | 1.003 (0.997, 1.009) | 0.335 |
| smoking | 0.987 (0.985, 0.989) | < 0.001 | 0.983 (0.981, 0.986) | < 0.001 | 0.984 (0.981, 0.987) | < 0.001 |
| sleep | 0.992 (0.987, 0.996) | <0.001 | 0.989 (0.985, 0.993) | < 0.001 | 0.991 (0.987, 0.996) | <0.001 |
| BMI | 0.998 (0.994, 1.001) | 0.192 | 0.998 (0.994, 1.001) | 0.233 | 0.996 (0.991, 1.002) | 0.207 |
| non-HDL-C | 0.992 (0.988, 0.995) | < 0.001 | 0.996 (0.992, 1.000) | 0.034 | 0.997 (0.993, 1.000) | 0.08 |
| blood glucose | 0.985 (0.981, 0.989) | < 0.001 | 0.992 (0.987, 0.997) | 0.002 | 0.993 (0.988, 0.998) | 0.004 |
| blood pressure | 0.990 (0.987, 0.993) | < 0.001 | 0.999 (0.995, 1.002) | 0.397 | 1.000 (0.996, 1.003) | 0.86 |
| Depression | 0.991 (0.986, 0.995) | <0.001 | 0.989 (0.984, 0.993) | < 0.001 | 0.992 (0.987, 0.997) | 0.002 |
| OR odds ratio, CI confdence interval. | | | | | | |
| Crude model, No adjustment for any potential influence factors. | | | | | | |
| Model 1, Adjusted for Sex, Age and Ethnic/race. | | | | | | |
| The scoring method for the common indicators of LE8 and LC9 is consistent. | | | | | | |
| Model 2, Adjusted for Sex, Age, Ethnic/race, Marital status, Family income-to-poverty ratio Education levels, lean body mass and Alcohol consumption status. | | | | | | |
| LE8 scoring algorithm consists of 4 health behaviors (diet (Healthy Eating Index, HEI), physical activity, nicotine exposure, and sleep duration) and 4 health factors (body mass index [BMI], non-high-density-lipoprotein cholesterol, blood glucose, and blood pressure) | | | | | | |
| LC9 scoring algorithm consists of 4 health behaviors (diet (Healthy Eating Index, HEI), physical activity, nicotine exposure (smoking), and sleep) and 4 health factors (body mass index (BMI), non-high-density-lipoprotein cholesterol (non-HDL-C), blood glucose, and blood pressure) and Depression. Therefore, this study presents LC9 levels from multiple dimensions. For example, the following four dimensions: quartile grouping (Q1, Q2, Q3, Q4), grouping based on the LE8 threshold (Low (0–49), Moderate (50–79), High (80–100)), LC9-per10 (continuous variable), LC9 (continuous variable). | | | | | | |
